# Comparison of the effectiveness of zero-profile device and plate cage construct in the treatment of one-level cervical disc degenerative disease combined with moderate to severe paraspinal muscle degeneration

**DOI:** 10.3389/fendo.2023.1283795

**Published:** 2023-12-06

**Authors:** Haimiti Abudouaini, Hui Xu, Junsong Yang, Mengbing Yi, Kaiyuan Lin, Sibo Wang

**Affiliations:** Department of Spine Surgery, Honghui Hospital, Xi’an Jiaotong University, Xi’an, Shanxi, China

**Keywords:** cervical paraspinal muscle, fatty infiltration, cross-sectional area, cervical disc degenerative disease, anterior cervical discectomy and fusion, stand-alone anchored cage

## Abstract

**Objective:**

Recent evidence indicates that cervical paraspinal muscle degeneration (PMD) is a prevalent and age-related condition in patients with cervical disc degenerative disease (CDDD). However, the relationship between surgery selection and post-operative outcomes in this population remains unclear. Consequently, this study aims to investigate the disparities in clinical outcomes, radiological findings, and complications between two frequently utilized anterior cervical surgical procedures. The objective is to offer guidance for the management of PMD in conjunction with CDDD.

**Methods:**

A total of 140 patients who underwent single-level anterior cervical discectomy and fusion (ACDF) at our department were included in this study. The patients were divided into three groups based on the severity of PMD: mild (n=40), moderate (n=54), and severe (n=46), as determined by Goutalier fat infiltration grade. The subjects of interest were those with moderate-severe PMD, and their clinical outcomes, radiological parameters, and complications were compared between those who received a stand-alone zero-profile anchored cage (PREVAIL) and those who received a plate-cage construct (PCC).

**Results:**

The JOA, NDI, and VAS scores exhibited significant improvement at all postoperative intervals when compared to baseline, and there were no discernible differences in clinical outcomes between the two groups. While the PCC group demonstrated more pronounced enhancements and maintenance of several sagittal alignment parameters, such as the C2-7 angle, FSU angle, C2-7 SVA, and T1 slope, there were no statistically significant differences between the two groups. The incidence of dysphagia in the zero-profile group was 22.41% at one week, which subsequently decreased to 13.79% at three months and 3.45% at the final follow-up. In contrast, the plate cage group exhibited a higher incidence of dysphagia, with rates of 47.62% at one week, 33.33% at three months, and 11.90% at the final follow-up. Notably, there were significant differences in the incidence of dysphagia between the two groups within the first three months. However, the fusion rate, occurrence of implant subsidence, and adjacent segment degeneration (ASD) were comparable at the final follow-up.

**Conclusion:**

For patients with one-level cervical disc degenerative disease combined with paraspinal muscle degeneration, both the zero-profile technique and PCC have demonstrated efficacy in ameliorating clinical symptoms and maintaining the postoperative sagittal balance. Although no significant disparities were observed between these two technologies in terms of complications such as adjacent segment degeneration and implant subsidence, the zero-profile technique exhibited superior performance over PCC in relation to dysphagia during the early stages of postoperative recovery. To validate these findings, studies with longer follow-up periods and evaluations of multilevel cervical muscles are warranted.

## Introduction

Cervical degenerative disc disease (CDDD) is a pathological condition characterized by the degeneration of intervertebral discs and subsequent degeneration of the adjacent intervertebral joints, resulting in detrimental effects on the surrounding essential tissues, including the spinal cord, nerve roots, sympathetic nerves, and vertebral arteries (1, 2). It typically presents as discomfort in the neck and shoulders, accompanied by stiffness, radiating sensations towards the head, pillow, or upper limbs. In more severe instances, it may lead to spasms in both lower limbs, hindered mobility, quadriplegia, and other related symptoms (3–5). According to a cohort study involving 47,560 patients, the incidence of CDDD is 13.1% (6), with a peak incidence in the fourth and fifth decades of life (7). Reportedly, total annual treatment costs for neck pain were estimated at $686 million in Netherlands and $800 million in China (8, 9).

In cases where conservative treatments prove ineffective, surgical intervention is advised for patients with CDDD. Since anterior cervical discectomy and fusion (ACDF) was first reported by Smith and Cloward in 1958, the procedure has gradually become one of the dominant surgical strategies in the treatment of single and double level CDDD (10), and previous literature revealed that ACDF indeed could provide favorable clinical outcomes and maintain the reconstruction of the cervical spine (11–13). It was reported that more than 100 000 patients receive this treatment in the US annually (3) and is projected to increase by more than 10% in the next 20 years (14). The ACDF with traditional plate-cage construct (PCC) with screws was the primary spinal surgical approach for addressing symptomatic cervical disc disease. This method offers several benefits, including the preservation or enhancement of cervical sagittal alignment and stability, improved fusion rates, decreased likelihood of graft extrusion, as well as reduced micromotion and subsidence of implanted cages (15–18). The placement of an anterior cervical plate in close proximity to the esophagus may lead to mechanical irritation and subsequent soft tissue swelling, ultimately resulting in secondary dysphagia (19–21). Consequently, the utilization of a novel stand-alone device featuring a zero-profile device has become prevalent in ACDF procedures as a means of mitigating plate-related complications. The zero-profile devices represent a viable substitute for traditional ACDF implants, as they have demonstrated efficacy in diminishing the incidence of adjacent segment degeneration, circumventing contact with the cervical spine’s anterior soft tissue, and potentially averting postoperative dysphagia (22). Nonetheless, scholars have discovered that patients who undergo ACDF with zero-profile device may encounter postoperative axial pain, loss of cervical curvature, and sagittal imbalance as a result of the absence of supplementary plate fixation (23).

The cervical paraspinal muscle (CPM) is a vital component of the dynamic spinal stabilization system, serving a critical function in preserving the stability and mobility of the neck (24). Through the recruitment of muscles and reflex responses of the nervous system, the neck muscles and tendons provide sufficient stability and regulate cervical motion. Recent research has revealed that degeneration of the CPM, in addition to bony structural alterations, is a significant contributor to persistent neck pain, sagittal imbalance, and the development of CDDD (25–27). Numerous patients with CDDD exhibit varying degrees of neck muscle degeneration, as evidenced by two abnormal indicators on MR images: a reduction in myofiber size (muscle atrophy) and an increase in fat deposition (fatty infiltration) (28). The co-occurrence of muscle atrophy and fatty infiltration is frequently observed due to the inclination of myosatellite cells to differentiate into adipocytes under pathological conditions (29). However, there is a paucity of research investigating the correlation between surgical selection and postoperative outcomes among patients afflicted with concurrent CDDD and CPM degeneration.

Given the limited availability of clinical data in this domain, a retrospective analysis was conducted to determine the more advantageous surgical approach for these patients. Specifically, the clinical and radiological outcomes of ACDF with zero-profile device versus ACDF with PCC system were compared. The findings of this study are anticipated to furnish valuable insights and practical recommendations for the management of CDDD patients with CPM degeneration in the foreseeable future.

## Materials and methods

### Study design

In our department, a retrospective review was conducted on patients who underwent single-level ACDF from C3 to C7 between January 2016 and May 2020. The decision to proceed with surgery was determined by a clinical presentation that was consistent with recent magnetic resonance imaging (MRI) findings of root or spinal cord compression. This study received approval from the Medical Ethics Committee of our hospital and all patients provided informed consent for the analysis of their clinical data.

### Inclusion and exclusion criteria for patients

The study’s inclusion criteria encompassed patients who exhibited radiculopathy or myelopathy stemming from single-level cervical disc disease, with corresponding magnetic resonance imaging evidence and a lack of response to conservative treatment for a minimum of six weeks. Additionally, eligible patients were over 18 years of age and had undergone ACDF utilizing either the zero-profile device or PCC system from C3 to C7. Furthermore, patients were required to have comprehensive postoperative anteroposterior and lateral X-rays, as well as clinical data, and had agreed to participate in at least one year of follow-up. The present study employed specific exclusion criteria, which included the following: cervical disc replacement (CDR) or hybrid surgery (CDR with ACDF); ACDF utilizing alternative types of devices; multilevel surgery; local or systemic infection; severe osteoporosis (T score < -2.5); pathological vertebral fracture or spinal deformity; allergy to the device material; ankylosing spondylitis; rheumatoid arthritis; or prior cervical spine surgery.

All surgical procedures were performed by one senior spinal surgeon in our department using a standard, right Smith- Robinson approach after the induction of general anesthesia (30, 31). The selection of the zero-profile device or PCC device was based on the patient’s condition and willingness. The zero-profile device group received a stand-alone cervical fusion implant (PREVAIL Medtronic Sofamor Danek, Memphis, TN) filled with a composite synthetic bone graft for ACDF, while the PCC group underwent ACDF using the VENTURE™ anterior cervical plate system (Medtronic Sofamor Danek, Memphis, Tennessee, USA) with an allograft.

### Clinical evaluation

The patients’ arm and neck pain were evaluated using the visual analogue scale (VAS), which measures pain on a scale of 0 to 10 points, with 0 representing the absence of pain and 10 representing the highest level of pain. The neck disability index (NDI) scores were used to evaluate the function of the neck. The NDI is a validated 10-item questionnaire, with each item rated on a 6-point scale (32). This study used the Chinese version of the NDI proposed by Wu et al. (33), which is specifically targeted at Chinese-speaking individuals with neck pain. It also uses a 6-point Likert scale that ranges from 0 (no disability) to 5 (complete disability) for each item. Disability ratings are assigned as follows: 0 to 4, no disability; 5 to 14, mild disability; 15 to 24, moderate disability; 25 to 34, severe disability; and above 34, complete disability. The Japanese orthopedic association (JOA) scores were used to assess the neurological status of patients with myelopathy, the myelopathy severity is considered mild if the JOA score is higher than 13, moderate if the JOA score ranges from 9 to 13, and severe if the JOA score is lower than 9 (34).

### Radiological evaluation

#### Evaluation of CPM degeneration

**Figure 1:**
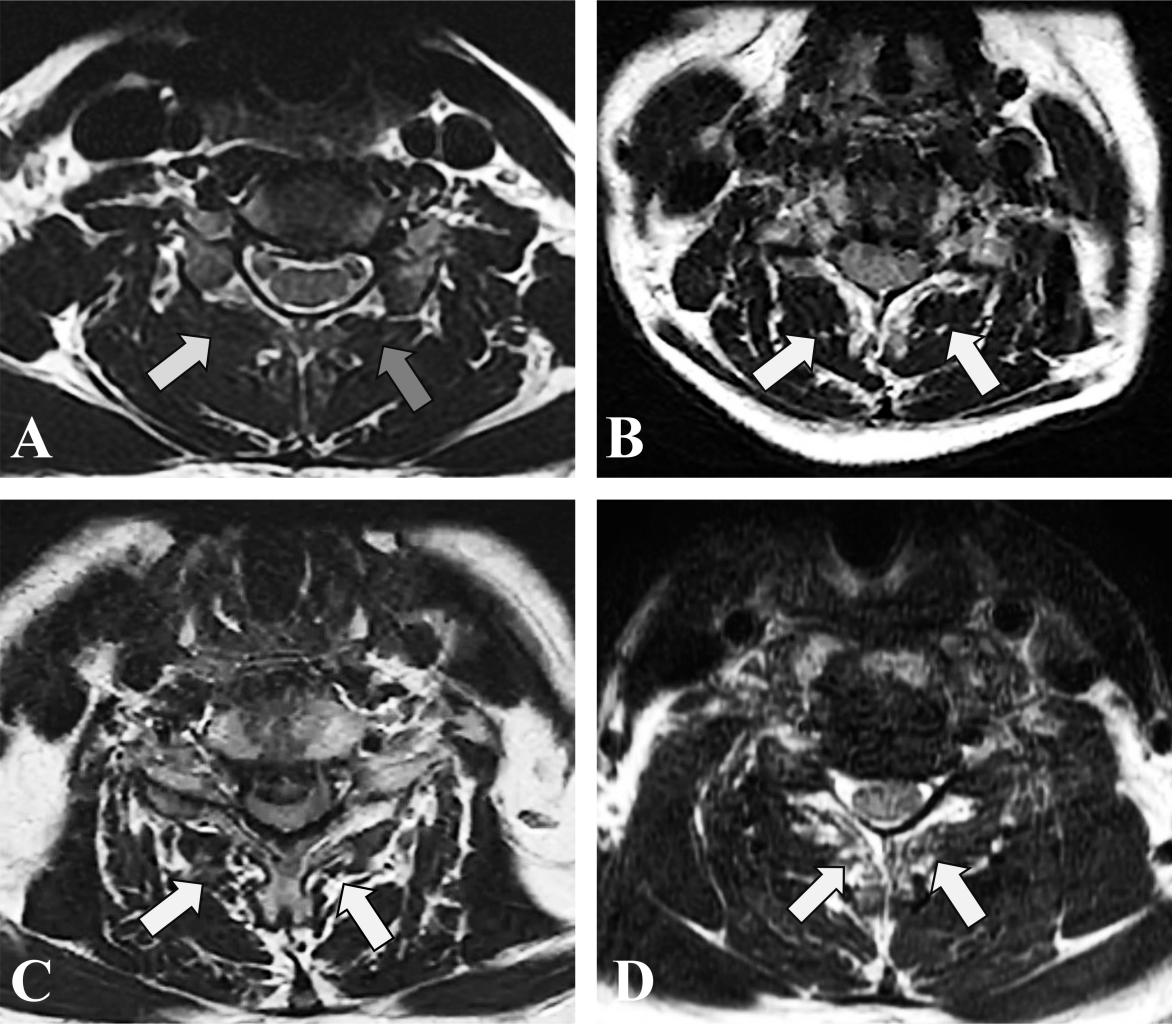
T2-weighted axial MRI section demonstrating fatty infiltration of muscle multifidus belly at C5/6. **(A)** Goutalier Grade 0 (white arrow), Goutalier Grade 1 (grey arrow); **(B)** Goutalier Grade 2. **(C)** Goutalier Grade 3. **(D)** Goutalier Grade 4.

**Figure 2:**
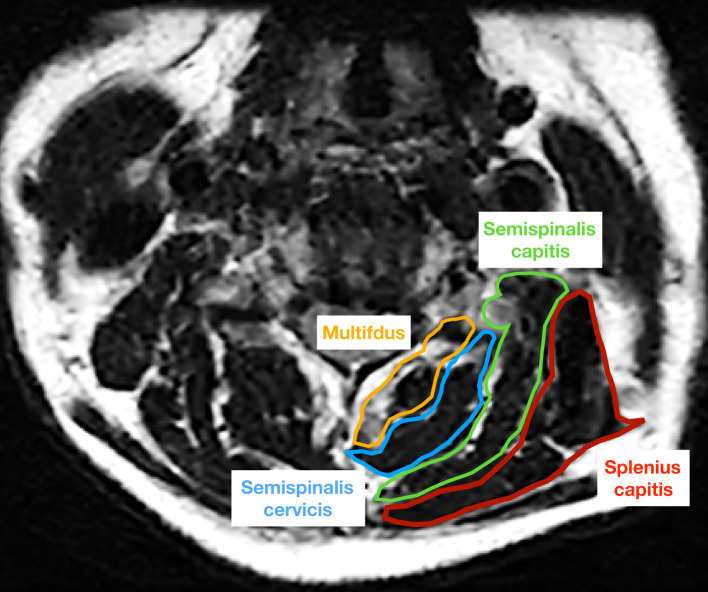
The cross-sectional area of multifdus (yellow), semispinalis cervicis (blue), semispinalis capitis (green), and splenius capitis (red) was measured on an axial T2 weighted image at the C5/6 level.

The study employed lateral X-ray images, computed tomography (CT) and MRI images to conduct radiological analysis. Prior to the operation, qualitative and quantitative evaluations of CPM were performed on an axial T2 weight section obtained from MRI. The degree of muscle fat infiltration at the C5/6 level was chosen as a representative measure of the cervical muscle, consistent with established practice in prior research (24, 35). The Goutallier classification was employed to assess the degree of fatty infiltration in the CPM prior to ACDF surgery, as documented in previous studies (24, 35). The Goutallier grading system utilizes scores ranging from 0 to 4, with 0 indicating the absence of visible fat streaks in the multifidus, 1 indicating minimal fatty streaks, 2 indicating a greater proportion of muscle than fat, 3 indicating equal amounts of fat and muscle, and 4 indicating a greater proportion of fat than muscle (**Table 1**, **Figure 1**). The multifidus muscles on both the right and left sides were evaluated separately, and the average of the scores was used for the final classification. Furthermore, the medial fascia boundaries of multifidus, semispinalis cervicis, semispinalis capitis, and splenius capitis at the C5-6 level on both sides were manually delineated using ImageJ software (v2.1.4.7; National Institutes of Health, USA) and quantified as the cross-sectional area (CSA) of each muscle **Figure 2**.

**Table 1:** The Goutallier fatty infiltration grade of paravertebral muscle degeneration.

Score	Severity	Fat infiltration
0	None	Absence of visible fat streaks
1	Mild	Minimal fatty streaks
2	Moderate	A greater proportion of muscle than fat
3	Moderate-Severe	Equal amounts of fat and muscle
4	Severe	A greater proportion of fat than muscle

#### Measurement of cervical sagittal alignment

**Figure 3:**
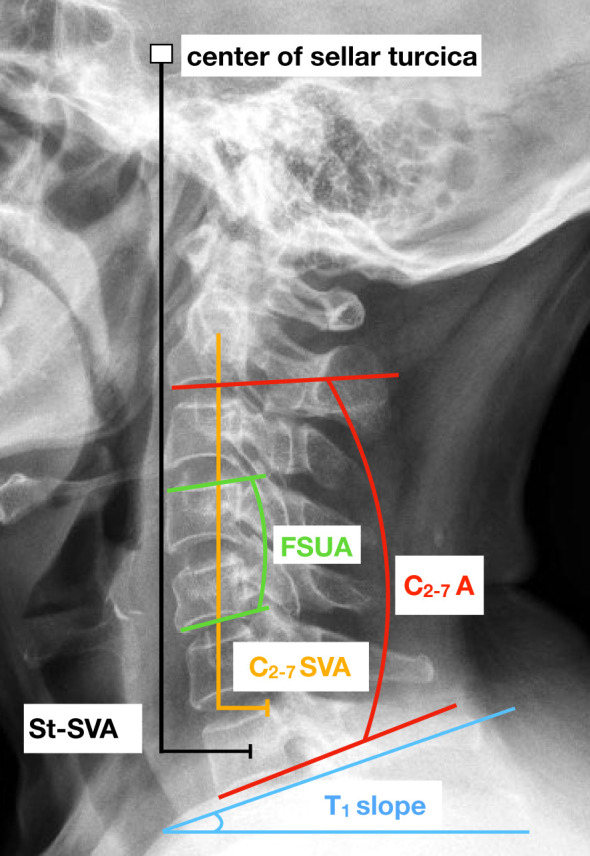
Lateral cervical spine radiograph with an illustration of key cervical sagittal alignment measurements. FSUA indicates the functional spinal unit angle. C_2-7_ A represents the C2-C7 angle. C_2-7_ SVA indicates the sagittal vertical axis and St-SVA indicates the center of the sella turcica – C7 sagittal vertical axis.

The present study recorded various parameters related to cervical spine, including cervical lordosis (CL), range of motion (ROM) of C2-C7, functional spinal unit angle (FSUA), sagittal vertical axis (C2-7 SVA), center of the sella turcica–C7 sagittal vertical axis (St-SVA), and T1 slope. The measurement techniques employed in this study were consistent with those described in previous literature (36, 37). Specifically, CL was determined by measuring the angle between the inferior margin of the C2 vertebrae and the inferior margin of the C7 vertebrae. The calculation of the FSU angle involved the utilization of the Cobb angle of the vertebrae adjacent to the intervertebral disc in question. The determination of the C2-C7 SVA was based on the measurement of the distance between the posterosuperior corner of C7 and the vertical line originating from the center of the C2 body. The center of the St-SVA was established as the distance between a plumb line originating from the center of the sellar turcica and the center of the C7 body. The T1 slope was defined as the angle formed between the T1 superior endplate and a horizontal line (**Figure 3**).

#### Complications

The study documented postoperative complications, namely dysphagia, adjacent segment degeneration (ASD) and implant subsidence. Dysphagia was evaluated by using the Bazaz grading system and the scores of the Bazaz grading system were ranked as follows: 0-none, 1-mild, 2-moderate and 3-severe, representing no episodes of swallowing problems, rare episodes of dysphagia, occasional swallowing difficulties with specific foods and frequent swallowing difficulties with most foods, respectively. ASD was characterized by the emergence of new or enlarged ossification of the anterior longitudinal ligament, new or increased narrowing of the disc space by more than 30%, new or obvious enlarging osteophyte formation, and endplate sclerosis (38). Implant subsidence pertains to a reduction in the height of the functional spinal unit (FSU) by more than 2 mm (39).

### Statistical analysis

The retrospective nature of the study predetermines the fixed sample size based on the available data. The statistical software SPSS version 22.0 (IBM Corp., Armonk, NY) was utilized for all analyses. Continuous variables were presented as the mean ± standard deviation, while categorical variables were presented as the rate and ratio index. The normality of the parameters was assessed through a Shapiro-Wilk test. To examine significant differences among the groups, one-way analysis of variance (ANOVA) and Kruskal–Wallis tests were conducted based on the distribution of variables. The Chi-squared test was employed for categorical variables. The preoperative and post-operative parameters were compared using either the paired t-test or the Wilcoxon signed-rank test. Statistical significance was determined by a p-value of less than 0.05.

## Results

### Patient demographic data

The study consisted of a total of 140 patients, with 80 patients (43 men and 37 women) in the zero-profile group and 60 patients (38 men and 22 women) in the PCC group. The average age of the zero-profile group was 51.54 ± 9.19 years, while the average age of the PCC group was 52.30 ± 10.96 years. Statistical analysis revealed no significant differences between the two groups in terms of age, sex, body mass index (BMI), bone mineral density (BMD), operated level, intraoperative time, intraoperative blood loss, median time of hospital stay, or follow-up period (**Table 2**).

**Table 2:** Comparison of general information between the zero-profile and the PCC group.

	zero-profile group (n=80)	PCC group (n=60)	p
Age (year)	51.59 ± 9.21	52.85 ± 11.206	0.164
Sex (male/female)	43/37	38/22	0.301
BMI (kg/m^2^)	23.51 ± 2.84	23.18 ± 2.95	0.895
Preoperative symptom			0.749
Radiculopathy	33	27	
Myelopathy	37	24	
Radiculopathy and Myelopathy	10	9	
Operated segment			
C3-C4	7	8	
C4-C5	12	9	
C5-C6	52	36	
C6-C7	9	7	
Cage height	6.33 ± 0.73	6.28 ± 0.69	0.531
Intraoperative time (minute)	119.88 ± 18.33	121.83 ± 20.27	0.274
Estimated blood loss (milliliter)	72.13 ± 21.51	75.00 ± 27.02	0.468
T-score	0.22 ± 1.41	0.15 ± 1.31	0.111
Follow-up (month)	18.60 ± 7.37	17.90 ± 7.12	0.352

### Degrees of fatty infiltration and grouping method

**Table 3** displays the categorization of all patients based on the Goutallier classification, with three distinct groups established. The fatty infiltration of the multifidus was graded as 0-1 Goutallier grade for group A, 1.5-2 Goutalier grade for group B, and 2.5-4 Goutallier grade for group C. The patient population for group A consisted of 40 individuals (23 male and 17 females; average age=45.06 ± 6.31years), while group B comprised 54 patients (31 male and 23 female; average age = 43.21 ± 7.53 years), and group C included 46 patients (24 male and 22 female; average age = 46.51 ± 7.6 years). The study revealed that in Group A, 22 patients (55.0%) underwent ACDF with a zero-profile implant, while 18 patients (45.0%) received ACDF with a PCC fixation. Similarly, in Group B, 31 patients (57.41%) underwent ACDF with a zero-profile implant, and 23 patients (42.59%) received ACDF with a PCC fixation. In Group C, 27 patients (57.41%) underwent ACDF with a zero-profile implant, while 19 patients (42.59%) received ACDF with a PCC fixation.

**Table 3:** Comparison of baseline information between the three groups.

	Group A (n=40)	Group B (n=54)	Group C (n=46)	p
Goutalier grade	0-1	1.5-2	2.5-4	
Degree of fat infiltration	Normal-Mild	Moderate	Severe	
Age (year)	50.67 ± 10.01	53.23 ± 10.45	51.06 ± 8.30	0.879
Sex (male/female)	22/18	31/23	28/18	0.857
BMI (kg/m^2^)	22.87 ± 2.71	23.50 ± 2.79	23.64 ± 3.11	0.420
Preoperative symptom				0.656
Radiculopathy	19	27	22	
Myelopathy	18	18	17	
Radiculopathy and Myelopathy	3	9	7	
Operated segment				0.102
C3-C4	9	2	3	
C4-C5	6	13	9	
C5-C6	20	33	28	
C6-C7	5	6	6	
Cage height	6.38 ± 0.74	6.26 ± 0.68	6.30 ± 0.73	0.738
Intraoperative time (minute)	128.50 ± 22.96	118.89 ± 19.80	126.20 ± 22.49	0.077
Estimated blood loss (milliliter)	71.75 ± 23.19	77.96 ± 26.66	76.30 ± 34.47	0.572
T-score	0.01 ± 1.43	0.29 ± 1.33	0.22 ± 1.36	0.602
Cross‐sectional area (mm^2^)				
Multifidus	227.13 ± 75.88	222.69 ± 72.74	219.54 ± 71.87	0.892
Semispinalis cervicis	318.23 ± 93.51	307.30 ± 97.27	302.72 ± 103.53	0.758
Semispinalis capitis	361.15 ± 139.18	349.13 ± 148.31	338.74 ± 111.55	0.744
Splenius capitis	416.58 ± 150.20	402.41 ± 138.47	395.26 ± 145.58	0.787
Follow-up (month)	18.45 ± 6.57	19.44 ± 8.87	16.83 ± 5.31	0.196
Implant type				0.941
zero-profile	22	31	27	
PCC	18	23	19	

### Mean CSA of the paraspinal muscles

The mean CSA of the multifidus muscle was found to be 227.13 ± 75.88 mm^2^ in group A, 222.69 ± 72.74 mm^2^ in group B, and 219.54 ± 71.87 mm^2^ in group C. Similarly, the CSA of the semispinalis cervicis muscle was 318.23 ± 93.51 mm^2^ in group A, 307.30 ± 97.27 mm^2^ in group B, and 302.72 ± 103.53 mm^2^ in group C. The CSA of the semispinalis capitis muscle was 361.15 ± 139.18 mm^2^ in group A, 349.13 ± 148.31 mm^2^ in group B, and 338.74 ± 111.55 mm^2^ in group C. Lastly, the CSA of the splenius capitis muscle was 416.58 ± 150.20 mm^2^ in group A, 402.41 ± 138.47 mm^2^ in group B, and 395.26 ± 145.58 mm^2^ in group C. No statistically significant differences were observed in the mean CSA of the paraspinal muscles across the three groups (**Table 3**).

### Zero-profile versus PCC

To explore the most effective treatment strategy for patients with CDDD and severe CPM degeneration (Goutallier grade 1.5-2 and Goutallier grade 2.5-4), we conducted a comparative analysis of therapeutic efficacy, sagittal parameters, and complications between the zero-profile group and the PCC group.

### Clinical outcomes

The preoperative clinical outcomes did not exhibit any significant differences between the two groups. However, all patients experienced a marked improvement in clinical symptoms following the operation. The mean JOA score increased in all groups, while the mean VAS score and NDI significantly decreased. Postoperative clinical outcomes did not demonstrate any significant differences between the zero-profile and PCC groups, as evidenced by **Table 4**.

**Table 4:** Comparison of clinical outcomes after ACDF with a zero-profile implant and PCC in patients with severe muscle degeneration.

	zero-profile group (n=58)	PCC group(n=42)	P
JOA scores			
preoperative	11.03 ± 1.34	10.88 ± 2.29	0.698
Last follow-up	15.62 ± 1.44	15.74 ± 1.40	0.684
VAS score			
preoperative	5.86 ± 1.12	6.10 ± 1.23	0.325
Last follow-up	1.72 ± 0.59	1.71 ± 0.60	0.271
NDI scores			
preoperative	28.14 ± 7.19	28.42 ± 7.01	0.843
Last follow-up	11.66 ± 5.27	12.44 ± 4.69	0.438

### Radiological findings

**Figure 4:**
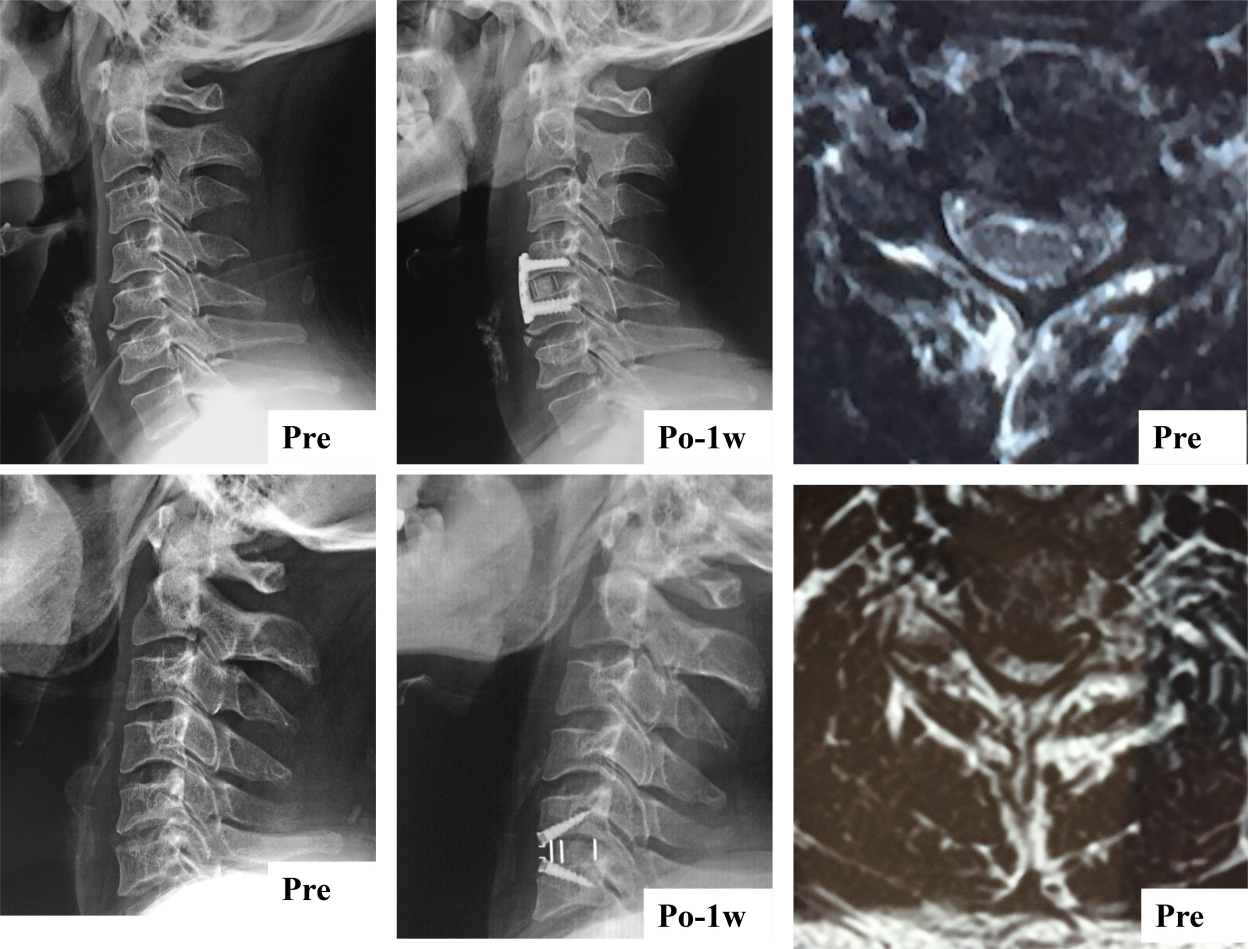
Sagittal parameters were improved whether cervical paraspinal muscle degeneration patients underwent ACDF using a Zero-profile device or conventional plate cage construct.

**Table 5** presents the imaging results, indicating that, with the exception of St-SVA at the final follow-up, the other sagittal alignment parameters were comparable across various time points **Figure 4**. Specifically, the St-SVA in the zero-profile group remained stable from 28.11 ± 7.17 mm pre-surgery to 26.45 ± 9.42 mm at the last follow-up, with a mean change value of -0.28 ± 6.65 mm. In contrast, the PCC group experienced a decrease in St-SVA from 27.86 ± 7.55 mm pre-surgery to 21.91 ± 8.61 mm at the last follow-up, with a mean change value of 2.09 ± 13.31 mm. Notably, there were significant differences between the groups in the St-SVA at the last follow-up (p=0.023).

**Table 5:** Comparison of radiographic assessments after ACDF with a zero-profile implant and PCC in patients with severe muscle degeneration.

Group	zero-profile group (n=58)	PCC group (n=42)	p
C_2-7_ angle (°)			
Preoperative	10.41 ± 7.92	9.39 ± 8.57	0.542
1 week	12.67 ± 7.63	14.67 ± 7.11	0.206
Last follow-upΔ	9.60 ± 7.25	12.00 ± 7.65	0.113
Δ C_2-7_ angle	3.07 ± 5.84	2.56 ± 4.86	0.649
FSU angle (°)			
Preoperative	1.12 ± 1.99	1.44 ± 1.98	0.346
1 week	3.20 ± 1.86	3.67 ± 1.42	0.173
Last follow-up	2.90 ± 1.98	3.51 ± 1.61	0.088
Δ FSU angle	- 0.32 ± 2.83	- 0.15 ± 1.55	0.734
C_2-7_ SVA (mm)			
Preoperative	19.83 ± 6.22	18.66 ± 6.04	0.351
1 week	22.41 ± 6.90	22.00 ± 5.02	0.748
Last follow-up	19.53 ± 6.56	20.33 ± 4.69	0.499
Δ C_2-7_ SVA	2.88 ± 8.79	1.68 ± 0.94	0.378
St-SVA (mm)			
Preoperative	28.11 ± 7.17	27.86 ± 7.55	0.628
1 week	23.75 ± 7.67	24.65 ± 8.96	0.412
Last follow-up	26.45 ± 9.42	21.91 ± 8.61	0.023*
Δ St-SVA	-0.81 ± 11.00	2.09 ± 13.31	0.236
T_1_ slope (°)			
preoperative	26.84 ± 6.88	27.31 ± 6.26	0.760
1 week	27.06 ± 5.64	26.72 ± 6.02	0.774
Last follow-up	26.54 ± 6.81	27.63 ± 6.46	0.421
Δ T_1_ slope	0.51 ± 7.05	-0.92 ± 7.23	0.327

* Indicates statistically significant differences (p<0.05).

### Complications

Over the course of several months following surgery, the overall occurrence of dysphagia exhibited a gradual decline in both groups. Specifically, the zero-profile group demonstrated a dysphagia incidence of 22.41% at one week, which decreased to 13.79% at three months and 3.45% at the final follow-up. In contrast, the plate cage group exhibited a dysphagia incidence of 47.62% at one week, which decreased to 33.33% at three months and 11.90% at the final follow-up. Notably, there were statistically significant differences between the groups in terms of dysphagia incidence within the initial three months (**Table 6**).

**Table 6:** Comparison of fusion rates and complications after ACDF with a zero-profile implant and PCC in patients with severe muscle degeneration.

	zero-profile group (n=58)	PCC group (n=42)	P
Fusion rate (%,n)	93.10% (54/58)	92.86% (39/42)	1.000
Subsidence (%,n)	12.07% (7/58)	9.52% (4/42)	0.757
Dysphagia(%,n)			
One week	22.41% (13/58)	47.62% (20/42)	0.010*
Three months	13.79% (8/58)	33.33% (14/42)	0.028*
Final follow-up	3.45% (2/58)	11.90% (5/42)	0.127
ASD (n,%)			
Superior	8.62% (5/58)	14.29% (6/42)	0.519
Inferior	15.52% (9/58)	19.05% (8/42)	0.788

*Indicates statistically significant differences (p<0.05).

## Discussion

Despite the widespread prevalence of cervical muscle degeneration, it has not garnered commensurate attention relative to the lumbar spine (40–44). He et al. observed a degeneration rate of 69.1% in the paraspinal muscles (Goutallier Grade ≥1.5) among patients with two-level cervical disc degenerative disease (24). They also identified a significant positive correlation between severe paraspinal muscle degeneration and postoperative sagittal balance disorder. Similarly, Wang et al. found that 67.33% (68/101) of patients with single-level cervical disc degenerative disease and severe fatty infiltration of paravertebral muscles experienced improved cervical sagittal alignment, which was comparable to those with strong cervical extensor muscles (39). The findings of our investigation align with prior research indicating that paraspinal muscle fatty degeneration can reach a prevalence of 71.43%. As a result, it is crucial to consider which ACDF procedure would be most advantageous for this demographic. Nevertheless, there is a dearth of literature on surgical decision-making for this cohort in previous studies.

The present study reports on the incidence of dysphagia in two groups, namely the zero-profile group and Plate group. The incidence of dysphagia in the zero-profile group was found to be 22.41% and 47.62% at one week, 13.79% and 33.33% at three months, and 3.45% and 11.90% at the final follow-up, respectively. It is noteworthy that all cases of dysphagia were mild or moderate and showed a decreasing trend over time. However, the increasing use of zero-profile implant was found to be associated with a higher risk of kyphotic deformity and poor dynamic stability due to the lack of anterior support, as reported in previous studies (15, 45). Lee et al. conducted a comparative analysis of postoperative retention and motion stabilization following ACDF utilizing three distinct implants (46). Their findings suggested that patients requiring robust postoperative motion stabilization should receive a plate-cage construct rather than a Zero-profile implant. Our investigation found that a single-level ACDF procedure utilizing a zero-profile or plate cage construct, with varying degrees of multifidus fatty infiltration, did not impact sagittal balance. One possible explanation for why no differences were seen between groups is that anterior surgery results in less obstruction to the paraspinal muscle (47). ACDF has the advantage of preserving the posterior muscles and avoiding injuring the posterior structures, such as the posterior ligaments, compared with posterior surgery. In the previous literature, most of the results that paravertebral muscle fat infiltration has an effect on cervical curvature and sagittal position parameters mainly focus on posterior cervical surgery. Preserving of the posterior structures in turn has an enormous impact on the mechanical stability of the cervical spine (48, 49). Our other hypothesis is that although cross-sectional area and degree of fat infiltration are now commonly used to evaluate paravertebral muscle degeneration, whether these indicators fully reflect paravertebral muscle function remains to be verified. Therefore, the indicators that can reflect the paravertebral muscle function should be explored in the future studies. However, the parameters of the final follow-up indicated that the sagittal vertical axis (St-SVA) was worse. Our hypothesis posits that a novel sagittal balance is established following single-level anterior cervical discectomy and fusion, thereby preserving the optimal horizontal plane of the preoptic system and maintaining the head axis. The degeneration of a solitary muscle within a singular segment may not be adequate to disrupt and exacerbate the state of equilibrium. Therefore, a comprehensive and extensive study of multiple-level ACDF procedures involving multiple cervical muscles, with long-term follow-up, is imperative. Furthermore, notable advancements have been achieved in the advancement of finite element (FE) models pertaining to cervical spine in recent decades. Consequently, employing the FE model of ACDF surgery to investigate the impact of cervical paravertebral muscle degeneration on postoperative biomechanical characteristics and sagittal balance emerges as one of the crucial and efficacious avenues for future scholarly inquiry (50).

According to estimations, cervical paraspinal muscles maintain approximately 80% of the mechanical stability of the cervical spine (51), which is essential for holding posture and stabilizing the head. The cervical paraspinal muscle is categorized into superficial, intermediate, and deep layers, forming a crucial dynamic equilibrium system of the cervical spine (52). The superficial layer of cervical paraspinal muscles comprises the trapezius, rhomboid, and levator scapulae muscles, while the intermediate layer primarily consists of the head clamp muscle, neck clamp muscle, and longest neck muscle, the deep layer primarily comprises the semi-spinous muscle and neck multifidus muscle. The flexion of the neck is primarily regulated by muscles such as the scalene muscle, longissimus capitis, and longissimus cervicalis, while extension is mainly controlled by the multifidus muscle, longissimus capitis, and suboccipital muscle. Lateral flexion is primarily governed by the head clamp muscle, neck clamp muscle, sternocleidomastoid muscle, and scalene muscle. Additionally, the lateral rotation of the neck is predominantly controlled by the sternocleidomastoid muscle, multifidus muscle, erector spine muscle, and head and neck clamp muscle (53–56).

The comprehensive examination and investigation of the molecular mechanism governing adipogenesis in muscle cells will enhance our comprehension of the interconversion between muscle and adipose tissues, the metabolic roles of muscle tissues, and the etiology of muscular disorders. Despite an incomplete understanding of the molecular mechanism underlying the muscle-adipose conversion, recent advancements have provided us with fresh perspectives on adipogenesis in muscle cells. To the best of our understanding, the development of muscle, bone, and adipose tissues encompasses a complex series of steps, beginning with the specification of a shared progenitor mesodermal cell towards a particular differentiation pathway, and subsequently leading to the manifestation of diverse terminal differentiation phenotypes (57). *In vitro* investigations have confirmed the pluripotent capacity of muscle-derived stem cells or precursor cells to differentiate in multiple directions (58, 59). To the best of our understanding, the development of muscle, bone, and adipose tissues encompasses a complex series of steps, beginning with the specification of a shared progenitor mesodermal cell towards a particular differentiation pathway, and subsequently leading to the manifestation of diverse terminal differentiation phenotypes (60). *In vitro* investigations have confirmed the pluripotent capacity of muscle-derived stem cells or precursor cells to differentiate in multiple directions. The multi-directional differentiation potential of muscle-derived stem cells or precursor cells has been demonstrated in *in vitro* studies (61). Additionally, lineage-tracing experiments have revealed that brown adipocytes, skeletal muscle cells, and dorsal dermal cells all originate from the same multi-potential progenitor cells derived from the central dermomyotome (62). Myoblasts have the potential to transdifferentiate into adipocytes or adipocyte-like cells under specific induction conditions (i.e., drug stimulation, cytokine treatment). Our findings also suggest that adipogenesis in muscle cells is prevalent among patients with cervical disc degenerative disease. Previous studies have reported the involvement of coding genes and non-coding genes, particularly miRNAs, in regulating the adipogenic transdifferentiation of myocytes (63). For example, miR-199a has been shown to regulate the transdifferentiation of C2C12 myoblasts by targeting the FATP1 gene (64). Moreover, the elimination of the interaction between slincRAD and the DNMT1 gene is anticipated to lead to impaired epigenetic regulation, thereby compromising the process of adipogenesis (65). Additionally, Qi et al. have documented that the lncRNA-GM43652 gene exhibits potential as a regulator of adipogenesis in muscle cells (66). However, this current level of understanding is insufficient to fully elucidate the regulatory functions of coding and non-coding genes in the process of transformation. Therefore, additional research is necessary to investigate the complex mechanisms involved in adipogenesis in muscle cells and to evaluate its association with prognostic outcomes in individuals suffering from cervical disc degenerative disease.

The current investigation is subject to certain limitations. Firstly, given that the study is retrospective, selection bias was unavoidable. Another limitation is the relatively small number of patients. Although we selected patients from January 2016 and May 2020, we limited the sample to only operations performed by the same doctor. Therefore, multicenter prospective design studies with a larger sample size are needed to verify our results. Secondly, the degree of muscle fat infiltration at the C5/6 level was exclusively chosen as a surrogate for the entirety of cervical muscle. While this approach has been employed in prior research (24, 35), it may not accurately reflect the actual mass of cervical muscles. Besides, although we measured muscle fat infiltration based on previously published reports, we acknowledge that potentially inherent radiographic imaging error might be a significant limitation. Another limitation of our study was the exact mechanism of the paraspinal muscle degeneration was did not explored. Additionally, the duration of the follow-up period was brief. Nonetheless, given that clinical outcomes, radiological parameters, and fusion rates stabilized and complications such as dysphagia and subsidence manifested within 12 months, the timeframe was deemed adequate for assessing short-term outcomes. However, a more extensive duration of follow-up would be required to examine the degeneration of adjacent segments and assess the long-term results.

## Conclusion

For patients with one-level cervical disc degenerative disease combined with paraspinal muscle degeneration, both the zero-profile technique and PCC have demonstrated efficacy in ameliorating clinical symptoms and maintaining the postoperative sagittal balance. Although no significant disparities were observed between these two technologies in terms of complications such as adjacent segment degeneration and implant subsidence, the zero-profile technique exhibited superior performance over PCC in relation to dysphagia during the early stages of postoperative recovery. To validate these findings, studies with longer follow-up periods and evaluations of multilevel cervical muscles are warranted.

## Data availability statement

The raw data supporting the conclusions of this article will be made available by the authors, without undue reservation.

## Ethics statement

The studies involving humans were approved by Medical Ethics Committee of Xi’an Jiaotong University. The studies were conducted in accordance with the local legislation and institutional requirements. The participants provided their written informed consent to participate in this study.

## Author contributions

HA: Writing – original draft. HX:. JY: Resources, Supervision, Writing – review & editing. MY: Methodology, Validation, Writing – review & editing. KL: Methodology, Software, Writing – original draft. SW: Formal analysis, Project administration, Resources, Supervision, Writing – review & editing.
